# Impact of Bi_2_O_3_ and ZrO_2_ Radiopacifiers on the Early Hydration and C–S–H Gel Structure of White Portland Cement

**DOI:** 10.3390/jfb10040046

**Published:** 2019-10-18

**Authors:** Qiu Li, Nichola J. Coleman

**Affiliations:** 1 State Key Laboratory of Silicate Materials for Architectures, Wuhan University of Technology, Wuhan 430070, China; qiu-li@whut.edu.cn; 2 Faculty of Engineering and Science, University of Greenwich, Chatham Maritime, Kent ME4 4TB, UK

**Keywords:** calcium silicate cement, endodontic bioceramic, Bi_2_O_3_, ZrO_2_, cement hydration, C–S–H gel, magic angle spinning nuclear magnetic resonance spectroscopy, calorimetry, filler effect

## Abstract

Bismuth oxide (monoclinic α-Bi_2_O_3_) and zirconium oxide (monoclinic ZrO_2_) are the most popular radiopacifiers in commercial Portland cement-based endodontic restoratives, yet their effects on the setting and hydration reactions are not fully understood. This study compares the impact of 20 wt.% of Bi_2_O_3_ or ZrO_2_ on the early hydration reactions and C–S–H gel structure of white Portland cement (WPC). Cement paste samples were hydrated at 37.5 °C prior to analysis by ^29^Si and ^27^Al magic angle spinning nuclear magnetic resonance spectroscopy at 3 h and 24 h, and transmission electron microscopy at 3 h. Initial and final setting times were determined using a Vicat apparatus and reaction kinetics were monitored by isothermal conduction calorimetry. Bi_2_O_3_ was found to prolong initial and final setting times and retard the degree of hydration by 32% at 24 h. Heat evolution during the acceleration and deceleration phases of the hydration process was reduced and the exotherm arising from renewed ettringite formation was delayed and diminished in the presence of Bi_2_O_3_. Conversely, ZrO_2_ had no significant impact on either setting time; although, it accelerated hydration by 23% within 24 h. Increases in the mean silicate chain length and the extent of aluminum substitution in the C–S–H gel were observed in the presence of both radiopacifying agents after 24 h relative to those of the unblended WPC. The Bi_2_O_3_ and ZrO_2_ particles remained intact within the cement matrix and neither bismuth nor zirconium was chemically incorporated in the hydration products.

## 1. Introduction

Hydraulic calcium silicate restoratives based upon Portland cement and its constituent dicalcium and tricalcium silicate phases are popular options for many endodontic procedures involving hard tissue repair and pulpal regeneration [1,2]. The properties and clinical applications of the current range of endodontic calcium silicate cements are extensively reported and reviewed in the recent scientific literature [1,2,3,4,5,6,7].

ISO 6876:2001 [8] and ANSI/ADA specification #57 [9] respectively stipulate that endodontic materials are required to possess a minimum radiopacity of 3 mm Al and a differential value of at least 2 mm Al between the cement and cortical bone. In order for commercial calcium silicate-based cements to comply with these standards, unreactive heavy metal oxide powders (such as Bi_2_O_3_, Zr_2_O, Ta_2_O_5_, Nb_2_O_5_, BaZrO_3_) are typically dry-blended into the formulation to achieve acceptable radiopacity [1,2,3,4,5,6,7]. Other candidate radiopacifiers (including CHI_3_, BaSO_4_, TiF_4_, CaWO_4_, Yb_2_O_3_, YbF_3_) have also been reported for experimental cements [10,11,12,13,14,15]; although, to date, the most commonly employed radiopacifying agents are bismuth (III) oxide (monoclinic α-Bi_2_O_3_) and zirconium (IV) oxide (monoclinic Zr_2_O) [1,2,3,4,5,6,7].

Both bismuth oxide and zirconium oxide are essentially chemically inert filler materials within the cement system, as neither directly engages in the hydration reactions of the Portland cement constituents [16,17,18]. Despite its lack of chemical reactivity, numerous studies have consistently observed that bismuth oxide prolongs both the initial and final setting times of Portland cement [11,15,19]. Conversely, zirconium oxide is variously reported to retard [19], accelerate [17], and also to have no impact on the setting reactions of Portland cements [11,15].

**Figure 1:**
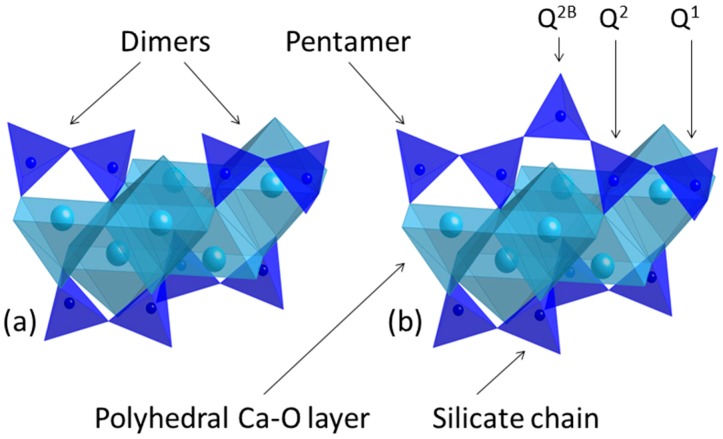
Calcium silicate layer structures of C–S–H gel showing (**a**) two dimers and (**b**) a pentamer unit (water molecules and hydroxyl groups are not shown).

The composition and setting reactions of Portland-based endodontic cements are widely described in the literature [6,16,17,20]. In brief, Portland cement consists of four impure hydraulic phases, alite (tricalcium silicate, Ca_3_SiO_5_), belite (β-dicalcium silicate, Ca_2_SiO_4_), aluminate (tricalcium aluminate, Ca_3_Al_2_O_6_) and ferrite (tetracalcium aluminoferrite, Ca_2_(Al/Fe)_2_O_5_), to which gypsum (CaSO_4_·2H_2_O) is added to regulate setting. The aluminate and ferrite phases initially react with water and gypsum to form needle-like crystals of ettringite (AFt, 6CaO·Al_2_O_3_·3SO_3_·32H_2_O) and its Fe-substituted counterpart, which subsequently decomposes to the thermodynamically more stable monosulphate (AFm, 4CaO·Al_2_O_3_·SO_3_·13H_2_O) phase [16,17,20]. Alite and belite react with water to form hexagonal crystals of portlandite (Ca(OH)_2_) and an adhesive nanoporous calcium silicate hydrate (C–S–H) gel of approximate composition (CaO)_1.7_SiO_2_(H_2_O)_4_, which constitutes ~70 wt.% of the hydrated cement matrix [21]. C–S–H is a highly disordered phase whose ideal structure comprises double layers of calcium oxide polyhedra linked on both sides to silicate chains, as shown in Figure 1.

On contact with water, the anhydrous monomeric (Q^0^) silicate tetrahedra of alite and belite are hydroxylated (Q^0^(H)), and dissolve and condense together to form dimers (Q^1^) that precipitate as colloidal C–S–H gel clusters [21,22]. The dimers in the C–S–H gel then merge together by the incorporation of bridging silicate (Q^2B^) or aluminate tetrahedra to form pentamers (Figure 1b). Aluminate tetrahedra are exclusively incorporated into the C–S–H gel in the bridging positions. Mid-chain silicate tetrahedra bonded to one aluminate tetrahedron are denoted as Q^2^(1Al) to distinguish them from mid-chain Q^2^ groups that are linked to two other silicate tetrahedra. Experimental and computational studies have shown that the number of condensed tetrahedra, *m*, in the aluminosilicate chain sequences obeys the empirical formula *m* = 3*n* + 1, where *n* is a positive integer [23,24]. Hence, the early silicate chain system in C–S–H gel is characterized by dimeric (*n* = 1) and pentameric (*n* = 2) units with the possible subsequent formation of octamers (*n* = 3), and also hendecamers (*n* = 4) in certain blended cements, at later stages [24,25].

The mean silicate chain length (MCL) of the C–S–H gel of Portland cement-based formulations influences the durability and performance of the matrix. Longer MCLs are generally associated with superior compressive strength and reduced solubility [24].

The principal objective of this present study was to compare the impact of 20 wt.% of micron-sized bismuth oxide or zirconium oxide on the early hydration reactions of white Portland cement. In particular, this study sought to understand the influence of these radiopacifiers on the structure of the C–S–H gel phase at a molecular level by evaluating the MCL and the extent of aluminum substitution (which have not been reported previously). Twenty wt.% (20 wt.%) radiopacifier was selected for this study, as this the most commonly used quantity of Bi_2_O_3_ or ZrO_2_ in both experimental and proprietary calcium silicate-based endodontic formulations and has been demonstrated to provide adequate radiopacity [1,2,6,10,16,17,20]. Cement paste samples were hydrated at 37.5 °C prior to analysis by ^29^Si and ^27^Al magic angle spinning nuclear magnetic resonance spectroscopy (MAS NMR) at 3 and 24 h, and transmission electron microscopy (TEM) at 3 h. Initial and final setting times were determined using a Vicat apparatus and reaction kinetics were monitored by isothermal conduction calorimetry to provide a comprehensive account of the influence of these popular radiopacifying agents on the early hydration chemistry of white Portland cement.

## 2. Results

### 2.1. Initial and Final Setting Times

The initial and final setting times for samples WPC, WPC-Bi, and WPC-Zr were obtained in accordance with ASTM C191-08 [26] and are listed in Table 1. These data demonstrate that the presence of 20 wt.% bismuth oxide significantly extends both the initial and final setting times of WPC by 112 min and 170 min, respectively; whereas, zirconium oxide has no impact on either setting time.

### 2.2. Isothermal Conduction Calorimetry

**Figure 2:**
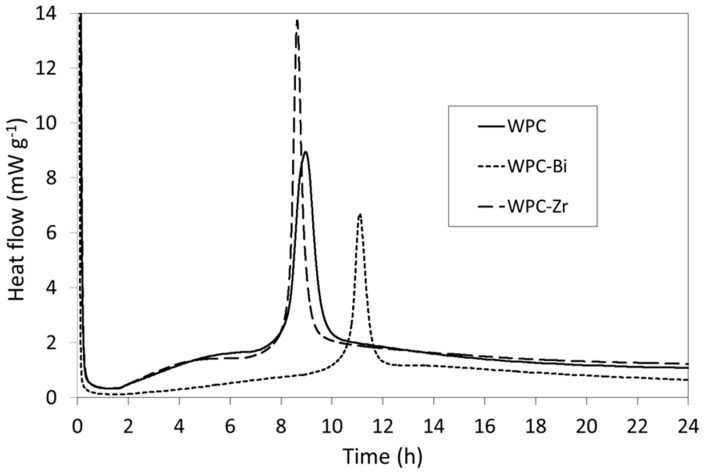
Rates of heat evolution from WPC, WPC-Bi, and WPC-Zr during hydration at 37.5 °C.

The rates of heat evolution per gram of cement powder for samples WPC, WPC-Bi, and WPC-Zr are plotted in Figure 2. Portland cements set via a complex sequence of exothermic reactions and the heat evolved during the first 24 h is largely governed by the formation of ettringite and the hydration of alite, which is in greater abundance and reacts more rapidly than belite [27]. In addition, the hydration of alite liberates more heat than that of belite, as their respective enthalpies of hydration are −517 J g^−1^ and −262 J g^−1^ (at 21 °C) [27]. For comparison, the complete hydration of the aluminate and ferrite phases in the presence of gypsum to form ettringite and its Fe-substituted analogue are respectively reported to release 1674 J g^−1^ and 418 J g^−1^ of heat (at 21 °C) [28].

Within the first few minutes of mixing WPC with water, the initial rapid evolution of heat is principally attributed to wetting, dissolution of free lime, sulphate and aluminate species, and to the precipitation of early hydrates (Figure 2) [27]. A dormant (induction) phase then ensues for approximately 1 h 45 min, during which the dissolution and precipitation reactions slow and heat production is reduced. The subsequent acceleration period is characterized by an increase in heat generation, which is determined by the rate of formation of the C–S–H gel product phase. The strongly exothermic signal observed after 8 h 47 min for sample WPC generates a maximum power of 9.09 mW g^−1^ and is attributed to renewed ettringite formation [27,28]. After this point, the deceleration phase is denoted by a steady decrease in heat evolution, which marks a decline in the hydration rate as the reactions become diffusion-controlled [27].

The heat evolution of WPC-Zr describes a similar pathway to that of the unblended WPC sample during the initial dissolution and precipitation phase, the induction period, and the beginning of the acceleration phase (Figure 2). In comparison, WPC-Zr gives rise to a sharper, more intense exotherm arising from renewed ettringite formation that reaches a maximum of 13.7 mW g^−1^ 15 min prior to that of WPC. As hydration continues into the deceleration phase, the heat released from WPC-Zr is ~0.16 mW g^−1^ greater than that of WPC. Conversely, following the induction period, the thermal profile of WPC-Bi presents a slow near-linear increase in the rate of heat evolution until approximately 10 h at the onset of renewed ettringite formation, which generates a maximum power of 6.65 mW g^−1^ at 11 h 7 min. The ongoing rate of heat evolution for WPC-Bi is then observed to be consistently lower than those of WPC and WPC-Zr.

As is typical for Portland cements, the initial and final setting times do not directly coincide with any specific events within the heat evolution profiles of samples WPC, WPC-Bi, and WPC-Zr. Although, unsurprisingly, both setting times for all three samples occur within the acceleration phase as the plastic cement matrix becomes rigid and early strength development begins [27].

### 2.3. ^27^Al MAS NMR Spectroscopy

^27^Al MAS NMR spectroscopy is used to discriminate among different aluminum co-ordination environments in Portland cement systems [29,30,31]. Tetrahedrally coordinated aluminum, such as that substituted into alite, belite, and C–S–H gel, gives rise to signals in the approximate chemical shift range of 100 to 50 ppm. Aluminum in octahedral environments (e.g., AFt and AFm phases) resonates in the range ca. 20 to −10 ppm; and signals arising from five-coordinate aluminum, which rarely occurs in cement systems, typically appear between 40 and 30 ppm [30,31].

**Figure 3:**
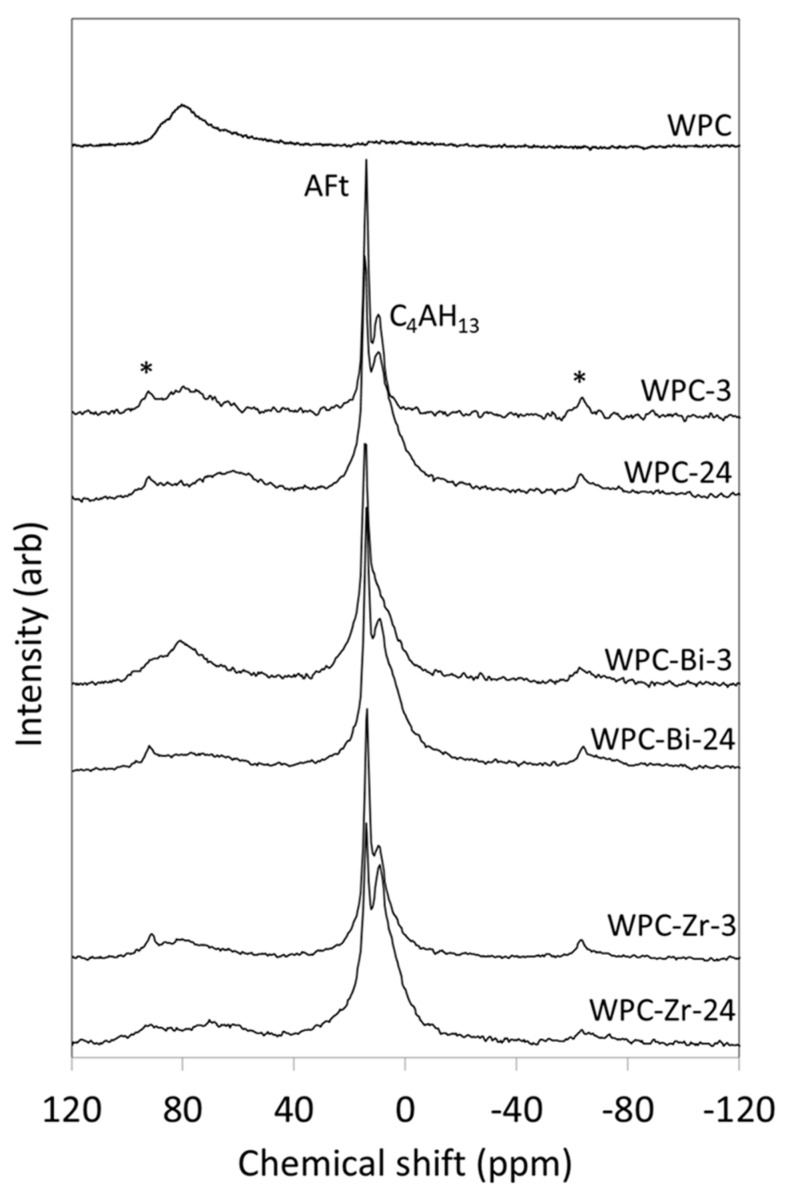
^27^Al MAS NMR spectra of anhydrous WPC and hydrated paste samples of WPC, WPC-Bi and WPC-Zr.

The ^27^Al MAS NMR spectrum of anhydrous WPC is shown in Figure 3 and comprises a broad resonance at ~82 ppm, which arises from tetrahedrally coordinated aluminum species substituted into alite and belite [31,32]. Signals from the aluminate and ferrite phases are not seen in the spectrum of anhydrous WPC due to extensive line-broadening. It should also be noted that the intensity of resonances of the various aluminum species are not in direct proportion to their relative concentrations owing to the quadrupolar nature of the ^27^Al nucleus [33].

Following 3 h of hydration, two unresolved peaks in the spectrum of WPC appear at 14.2 ppm and 9.8 ppm, which are respectively assigned to octahedrally coordinated aluminum in ettringite (AFt) and tetracalcium aluminate hydrate (C_4_AH_13_) (Figure 3) [31]. The latter phase forms by hydration of the aluminate phase when the supply of sulphate ions becomes limited. Within this timeframe, the intensity of the signal at ~82 ppm is markedly reduced, and after 24 h, it is partially replaced by a broad resonance at ~62 ppm arising from the incorporation of aluminate tetrahedra in the bridging positions of the C–S–H gel phase. ‘Spinning side bands’ are harmonic artefacts that result from the modulation of the magnetic field at the spinning frequency and are denoted by asterisks.

The ^27^Al MAS NMR spectra of the hydrating WPC-Zr sample resemble those of unblended WPC, in that the resonance at ~82 ppm is significantly reduced by 3 h and is accompanied by partially resolved signals for ettringite and tetracalcium aluminate hydrate at 13.9 ppm and 9.3 ppm, respectively (Figure 3) [31]. Within 24 h, a very broad resonance also develops at ~65 ppm from bridging aluminate tetrahedra in the C–S–H gel phase [31].

The incorporation of bismuth oxide is seen to slow the initial dissolution of the guest aluminate ions from alite and belite as the signal at ~82 ppm remains largely intact after 3 h (Figure 3) [31]. The early formation of the calcium (sulpho)aluminate hydrate phases is also affected by the presence of bismuth oxide, as the tetracalcium aluminate hydrate signal appears as a shoulder on the ettringite resonance after 3 h, rather than a distinct, yet partially-resolved peak, indicating that its development is retarded.

Other than the early retarding effect of bismuth oxide, there is no evidence to indicate that the incorporation of either radiopacifier in the cement system has any direct chemical impact on the formation of the calcium (sulpho)aluminate hydrate phases.

### 2.4. ^29^Si MAS NMR Spectroscopy

The chemical shifts of resonances in single pulse ^29^Si MAS NMR spectra reflect the degree of polymerization of the silicate species from which they arise and the signal intensities are proportional to their relative concentrations [29,30,31,33]. Isolated Q^0^ species (such as those in alite and belite) give rise to signals in the range −65 to −75 ppm; dimeric and chain-end Q^1^ groups resonate between −78 and −82.5 ppm; and signals in the range −84 to −87.5 ppm are assigned to mid-chain Q^2^ species [29,30,31,33]. Substitution of a neighboring silicate for aluminate tetrahedron (in the C–S–H bridging positions) increases the chemical shift by ~5 ppm, such that mid-chain Q^2^(1Al) species resonate ca. −81 to −83 ppm.

**Figure 4:**
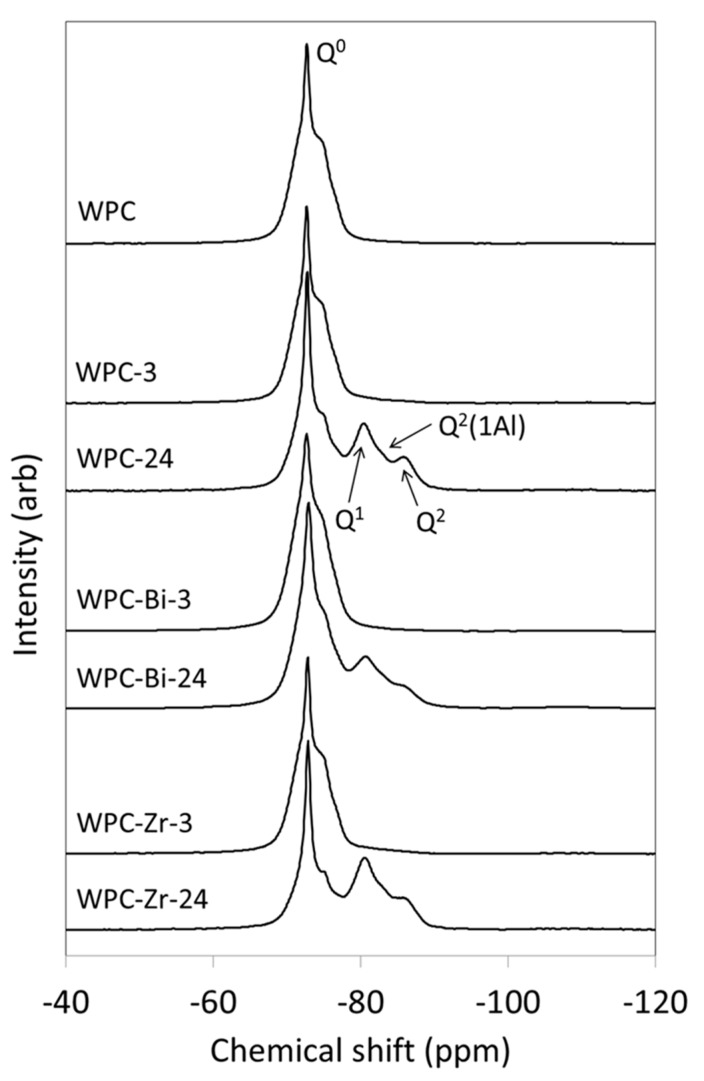
^29^Si MAS NMR spectra of anhydrous WPC and hydrated paste samples of WPC, WPC-Bi, and WPC-Zr.

The ^29^Si MAS NMR spectrum of anhydrous white Portland cement used in this study is shown in Figure 4. This spectrum comprises a sharp signal at −72.5 ppm from the single Q^0^ tetrahedral silicate environment in belite and an underlying broad resonance spanning the range −67 to −78 ppm, which is attributed to the nine crystallographically distinct Q^0^ silicate species in alite [32]. For all samples, WPC, WPC-Bi, and WPC-Zr, resonances from Q^1^, Q^2^, and Q^2^(1Al) appear at the expense of the signal from alite as hydration proceeds (Figure 4). In all cases, the rate of disappearance of the belite signal is slower than that of alite, owing to the difference in the hydration kinetics of these two phases. Qualitative comparison of the ^29^Si MAS NMR spectra after 24 h indicates that the rates of consumption of alite and formation of the Q^1^, Q^2^, and Q^2^(1Al) hydration products in sample WPC-Bi are slower than those of WPC and WPC-Zr.

### 2.5. Structure of C–S–H Gel

**Figure 5:**
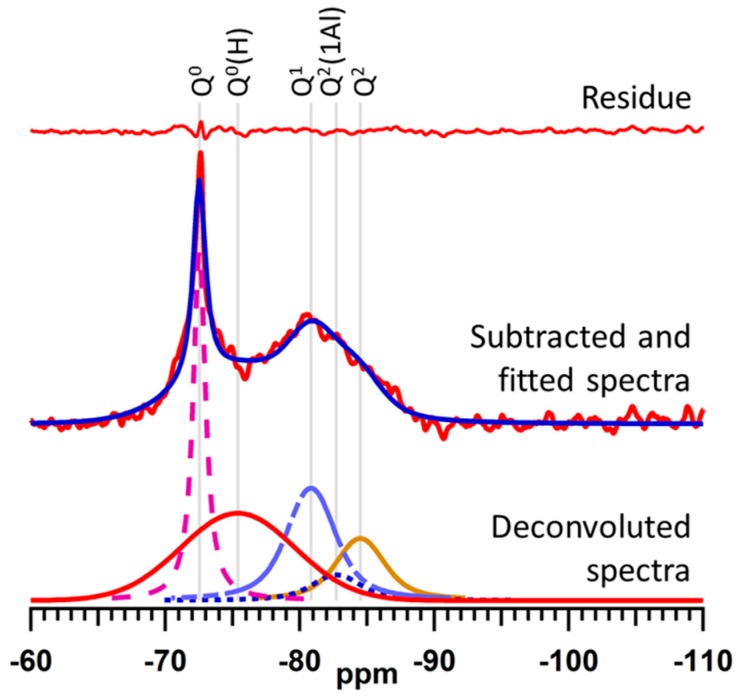
Subtracted, fitted, and deconvoluted ^29^Si MAS NMR spectra of hydrating. ZrO_2_-blended cement (WPC-Zr-3).

The broad signal of the residual anhydrous alite phase obscures the region of the ^29^Si MAS NMR spectrum in which the resonances of the early Q^0^(H) and Q^1^ hydration products appear. To address this problem, the ^29^Si MAS NMR spectra of the hydrating cement samples were analyzed by a method reported by Love et al. [29], in which the signals of unreacted alite are subtracted from the spectra prior to deconvolution and quantitative analysis. As an example, the subtracted, deconvoluted, and calculated spectra of the sample blended with zirconium oxide after 3 h of hydration (WPC-Zr-3) are shown in Figure 5. The residue (i.e., the difference between the subtracted and calculated spectra) is plotted above the spectra.

The relative abundance of hydrated Q^n^ species, the degree of hydration, the mean aluminosilicate chain length (MCL), and the Al/Si ratio of the C–S–H gel in WPC, WPC-Bi, and WPC-Zr after 3 h and 24 h of hydration are listed in Table 2. The extent of hydration for all samples is a few percent within the first 3 h. No significant differences in the degrees of hydration among the samples were found, as the error limits for this parameter are estimated to be approximately ±0.5%; however, notable differences between the C–S–H gel structure of WPC-Bi-3 and those of WPC-3 and WPC-Zr-3 were observed at this time-point. After 3 h, the silicate chains of WPC-Bi-3 were entirely composed of dimers (i.e., MCL = 2) and there were no detectable aluminate tetrahedra present in the bridging positions. In comparison, the C-S-H gel structure of WPC-3 comprised ~33% dimers and ~77% pentamers with ~6% aluminum substitution; and similarly, the aluminosilicate chain system of WPC-Zr-3 was formed from ~40% dimers and ~60% pentamers with ~3.5% aluminum substitution.

Marked variations in the degree of hydration and aluminosilicate chain structure among the three samples were observed after 24 h (Table 2). These data confirm that, zirconium oxide accelerates the extent of hydration from 42.2% (for unblended WPC) to 51.9% by 24 h; and also, that bismuth oxide retards hydration within this timeframe (to 28.6%). Despite the diminished hydration rate, the MCL of sample WPC-Bi-24 (4.6) is significantly longer than those of the other samples, indicating a predominance of pentameric units, and possibly octameric units, with a high proportion of aluminate substitution within the C–S–H gel.

### 2.6. Transmission Electron Microscopy (TEM)

**Figure 6:**
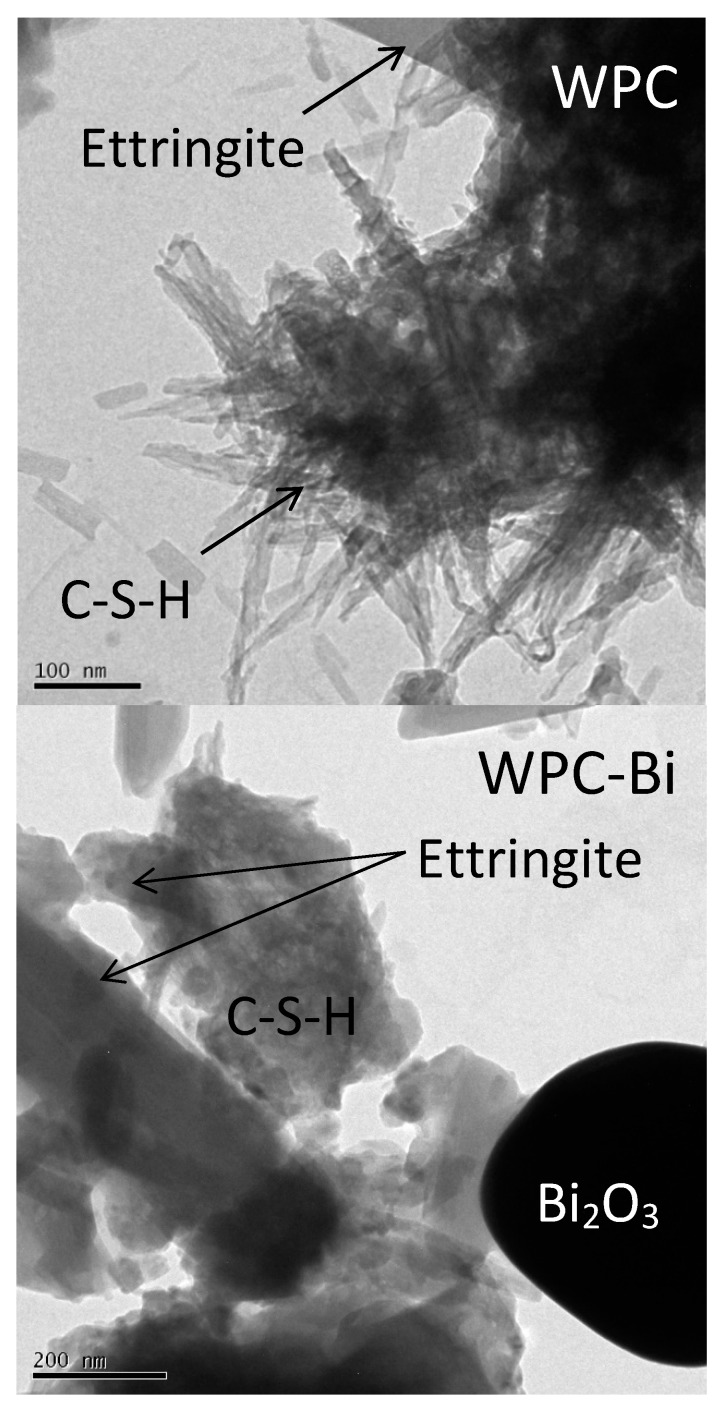

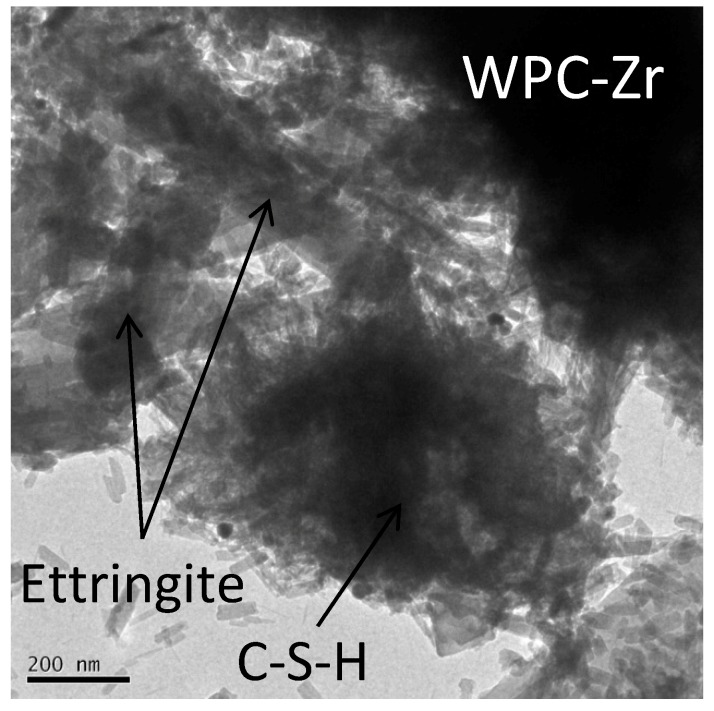
TEM images of WPC-3, WPC-Bi-3, and WPC-Zr-3.

TEM bright field images of WPC-3, WPC-Bi-3, and WPC-Zr-3 are shown in Figure 6. The morphologies of the early hydration products were similar for all samples irrespective of the nature of the radiopacifier. Fine fibrillar C–S–H gel of approximately 20 nm in width is found intermixed with lath-like crystals of ettringite (~100–200 nm in width) after 3 h of hydration. Zirconium oxide was found to be in intimate contact with the early C–S–H gel, whereas the surface of the bismuth oxide particles was largely unpopulated by the hydration products. TEM analysis also confirmed that the bismuth oxide and zirconium oxide particles remain intact within the cement matrix and that neither zirconium nor bismuth is transferred to the hydration products.

## 3. Discussion

Formulations based on Portland cement and its constituent hydraulic tricalcium and dicalcium silicate phases require the addition of radiopacifying agents in order to comply with the international standards for endodontic materials [1,2,3,4,5,6,7,8,9]. Bismuth oxide and zirconium oxide are the most popular radiopacifiers in commercial Portland cement-based endodontic restoratives, yet their effects on the setting and hydration reactions are not well understood at a molecular level and are currently disputed. Comprehensive knowledge of the impact of additives on the chemistry of calcium silicate endodontic cements is essential to the successful future development and applications of these materials. In the present study, white Portland cement was selected as a representative model for the investigation of the effects of micron-sized bismuth oxide and zirconium oxide on early hydration chemistry at 37.5 °C (i.e., body temperature), as many commercial endodontic cements are based on this material [1,2,3,4,5,6,7].

The findings of this research indicate that the incorporation of 20 wt.% zirconium oxide particles has no significant impact on the initial and final setting times of the white Portland cement (Table 2), both of which occur within the accelerating phase of the hydration profile (Figure 2). However, the maximum rate of heat generated during renewed ettringite formation is higher in the presence of zirconium oxide, as is the ongoing heat evolved during the deceleration phase (Figure 2).

Within the first 3 h, the degrees of hydration of the ZrO_2_-blended and unblended pastes are similar, although by 24 h the extent of hydration of WPC-Zr (~52%) exceeds that of WPC (~42%). Between 3 and 24 h, the mean chain length of the C–S–H gel of the unblended sample decreases from 4.0 to 3.7, with a modest concomitant increase in the Al/Si ratio, indicating that the proportion of dimers in the aluminosilicate chains increases. Conversely, within this timeframe, the MCL of WPC-Zr increases from 3.8 to 4.0 and is accompanied by a significant increase in bridging aluminate tetrahedra that effect the formation of pentamers.

In general, MCL is expected to increase with an increasing proportion of reactive aluminum in the hydrating system (as bridging aluminate tetrahedra link existing silicate units to form longer chains) and also to increase with temperature and time [25,29,31,34]. Modest decreases in MCL during the early stages of hydration, such as that observed here for unblended WPC, are uncommon but have been documented previously [31].

The MCLs for WPC and WPC-Zr found in this study are higher than those in the literature for white Portland cements of similar composition hydrated at ambient temperatures (although with varying water:cement ratios). For comparison, after 24 h, MCLs of 2.95 at 20 °C (w:c = 0.5) [25,31] and 3.51 at 25 °C (w:c = 0.425) are reported [34]. The superior MCL values, observed here at 37.5 °C, are attributed to a number of temperature-related phenomena that affect the development and structure of the C–S–H gel phase in Portland cements. Principally, higher curing temperatures are known to accelerate the rate of hydration and also to enhance the solubility of the aluminate hydrate phases, which increases the availability and subsequent uptake of aluminate tetrahedra by the C–S–H gel [28,34]. Elevations in temperature also reduce the solubility of calcium hydroxide (as this compound exhibits retrograde solubility), and hence, decrease the concentration of dissolved Ca^2+^ ions in the pore solution. Experimental and computational studies have demonstrated that lower Ca/Si ratios give rise to higher MCLs in Portland cements [22,34]. In fact, this is a common occurrence in many calcium silicate systems, as calcium ions are widely acknowledged to depolymerize the silicate structures of glasses, minerals, and ceramics [22,35].

The observed accelerating effect of zirconium oxide between 3 and 24 h into hydration, which is evidenced by a higher degree of hydration and longer aluminosilicate chains, confirms the findings of a number of previous studies and is attributed to the ‘filler effect’ [17,18]. The incorporation of various micron- and nano-sized metal oxides and carbonates, such as CaCO_3_, SiO_2_, Al_2_O_3_, ZrO_2_, and TiO_2_, in Portland cement systems is known to accelerate early hydration reactions [17,18,36,37]. This effect derives from the availability of nucleation sites on the surfaces of the filler particles which promote the precipitation and development of the early hydration products. The presentation of favorable heterogeneous nucleation sites on the filler also diverts the nucleation of hydration products from the surface of the Portland cement particles (which can provide a barrier to the ongoing dissolution of the anhydrous phases and impede the progress of the hydration) [36].

The extent of the filler effect depends upon chemical structure and surface properties (i.e., processing history), and is generally found to be inversely related to particle size [36,37]. In addition to a diffuse negative surface charge, which is expected under the highly alkaline conditions encountered during initial hydration, metal oxide and carbonate compounds may also present specific broken-bond sites or functional groups that act as nucleation centers.

In all probability, the inconsistencies among the various reports that zirconium oxide is potentially retarding [19], accelerating [17,18], and that it also has no impact on the setting reactions of Portland cements [11,15] are likely to have arisen from differences in particle size and processing history of the ZrO_2_. Furthermore, variations in the water:cement ratio of the formulations reported in the literature may also have contributed to these discrepancies.

In this study, the incorporation of 20wt.% bismuth oxide particles was found to significantly prolong both the initial and final setting times of the white Portland cement (Table 2), and also to cause marked changes to the heat evolution profile during the first 24 h of hydration (Figure 2). Following the induction period, a slow near-linear increase in heat evolution replaces the characteristic curve usually associated with the onset of the acceleration phase and the exotherm arising from renewed ettringite formation is delayed and diminished (Figure 2). The ongoing heat evolved during the deceleration phase is also lower than that of the unblended WPC paste (Figure 2).

Within the first 3 h, the degrees of hydration of the BiO_2_-blended and unblended pastes are similar, although the structures of the early C–S–H gel phases differ. Unlike the C–S–H gel of WPC-3, the silicate chain system of WPC-Bi-3 is exclusively composed of dimers (MCL = 2), with no bridging aluminate tetrahedra. The corresponding ^27^Al spectrum of sample WPC-Bi-3 demonstrates that the early dissolution of aluminate species from alite and belite is reduced in the presence of bismuth oxide, which restricts their availability to participate in C–S–H gel formation (Figure 3). The development of the calcium (sulpho)aluminate hydrate phases was also delayed in this sample (Figure 3).

After 24 h, the extent of hydration of WPC-Bi (~29%) is retarded with respect to that of WPC (~42%); although by this time, the belated release of aluminate species from alite and belite (Figure 3) is seen to promote the polymerization of the aluminosilicate chains in the C–S–H gel phase via bridging aluminate tetrahedra, which drastically increase both MCL and Al/Si relative to those of the unblended paste (Table 2). The observed retarding effect of bismuth oxide is unusual, since the incorporation of chemically-inert finely divided metal oxide compounds tends to accelerate the hydration reactions of Portland cements via the filler effect (as described previously) [17,18,36,37].

The extent of the filler effect is strongly influenced by the surface charge properties of the filler, which determine the specific mechanisms of C–S–H nucleation and growth [36]. Strong interactions between Ca^2+^ ions and the filler surface (such as those observed for CaCO_3_) facilitate the formation of stable nuclei that can grow to form macroscopic precipitates of C–S–H. Conversely, weak interactions between Ca^2+^ ions and the filler (e.g., those of SiO_2_) cause the adsorbed Ca^2+^ ions to diffuse back into solution, thus reducing the tendency to form stable nuclei [36].

Under the highly alkaline conditions encountered within the early cement matrix, bismuth oxide is anticipated to exhibit a diffuse negative surface charge as the pH far exceeds its point of zero charge (pH ~4) [38]. This negative surface charge would be expected to initiate the filler effect by weakly attracting Ca^2+^ ions via electrostatic interactions. However, bismuth oxide is a semiconductor, and hence, it is tentatively suggested that the mobile charge (i.e., electrons and positive holes) within the lattice may prevent stable localized electrostatic interactions between the surface and adsorbed Ca^2+^ ions [39]. The failure of a filler to support the formation of stable nuclei inevitably directs the precipitation of the early hydration products to the surface of the Portland cement particles, thus inhibiting the initial progress of hydration.

Additives that increase MCL are usually associated with superior microstructures, enhanced compressive strength, and reduced solubility [24]; however, bismuth oxide is generally acknowledged to have a deleterious impact on the porosity, mechanical strength, and durability of the cement matrix [11,40]. Despite the high MCL observed in the presence of bismuth oxide, which is expected to give rise to improved properties, poor bonding between this filler and the hydrated cement phases could potentially account for the reported deterioration in microstructural, mechanical, and chemical behavior.

The findings of this study support the current body of research which indicates that zirconium oxide is a potentially superior radiopacifying agent to bismuth oxide in Portland cement-based endodontic restoratives. From a clinical perspective, the extended initial and final setting times observed in the presence of bismuth oxide are a particular disadvantage. Understanding the impact of radiopacifiers on the setting reactions and hydration chemistry of calcium silicate cements is essential to the development of the next generation of these materials. In this respect, ^29^Si and ^27^Al NMR spectroscopy is an invaluable technique for elucidating the structural evolution of the principal C–S–H gel product on a molecular scale. In addition to understanding the impact of radiopacifiers and other admixtures on the hydration of calcium silicate-based endodontic cements, solid state NMR could also provide valuable information on the effect of the clinical environment (e.g., exposure to various etching agents, irrigants, and physiological fluids) on these materials.

## 4. Materials and Methods

### 4.1. Materials

The WPC used in this study was supplied by Lafarge (Gravesend, UK) and is commercially available as Snowcrete. Its oxide and Bogue phase compositions were provided by the manufacturer and are listed in Table 3. Bismuth oxide (>99% purity, 2 μm diameter) and zirconium oxide (99% purity, 5 μm diameter) were purchased from Sigma-Aldrich (Gillingham, UK).

### 4.2. Initial and Final Setting Times

WPC paste samples were prepared by manually mixing 10 g of cement with 3.75 g of distilled water for 5 min. Samples blended with 20 wt.% bismuth oxide or zirconium oxide (viz. WPC-Bi and WPC-Zr, respectively) were prepared similarly with partial replacement of 2 g of the WPC by the metal oxide radiopacifier at a water:solid ratio of 0.3 (i.e., a water:cement (w:c) ratio of 0.375). Initial and final setting times of the cement pastes were determined by the method described in ASTM C191-08 using a manual Vicat apparatus [26]. Setting data for each cement were collected in duplicate and were subjected to a two-tailed *t*-test at *p* = 0.05.

### 4.3. Isothermal Conduction Calorimetry

The rates of heat evolution during hydration of samples WPC, WPC-Bi, and WPC-Zr, were measured by isothermal conduction calorimetry using a Thermometric 2277 TAM calorimeter (Thermometric AB, Stockholm, Sweden) at 37.5 °C (i.e., body temperature). In duplicate, ~0.05 g of accurately weighed cement paste was placed in the calorimeter immediately after mixing. Power data were collected every second for 24 h. The rate of heat evolution per unit gram of cement powder was then calculated by dividing the power data by the original mass of white Portland cement in the paste.

### 4.4. ^27^Al and ^29^Si Magic Angle Spinning Nuclear Magnetic Resonance Spectroscopy

Cement paste samples WPC, WPC-Bi, and WPC-Zr were prepared in duplicate by manually mixing WPC, the required quantity of radiopacifying metal oxide and deionized water for 5 min at a water:cement ratio of 0.375 by mass (as described in Section 4.2). The samples were then compacted and hermetically sealed into polypropylene tubes and cured at 37.5 °C for 3 and 24 h. Prior to analysis, the hydration reactions were stopped by solvent exchange with propan-2-ol. This was achieved by immersion of 2 mm fragments of the pastes in four consecutive 50 cm^3^ washings of propan-2-ol in a sonic bath for 30 min. The samples were then dried to constant mass in a vacuum desiccator at room temperature.

MAS NMR spectra of anhydrous WPC cement powder and the hydrated samples were collected on a JEOL JNM-ECX 300 MHz spectrometer (JEOL, Tokyo, Japan). Single pulse ^27^Al MAS NMR spectra were obtained with a pulse delay of 0.5 s, an acquisition time of 0.01024 s, and 7000 scans. Single pulse ^29^Si MAS NMR spectra were obtained with a pulse delay of 5 s, an acquisition time of 0.02048 s, and a minimum of 70,000 scans. All spectra were collected with a spin rate of 6 kHz. ^27^Al and ^29^Si chemical shifts were referenced to tetramethylsilane (TMS) and the aluminum hexaquo ion [Al(H_2_O)_6_]^3+^, respectively. The raw data (i.e., free induction decay (FID) files) were processed using Delta software (JEOL, Tokyo, Japan) to obtain spectra, which were then analyzed and deconvoluted using Igor Pro software (WaveMetrics Inc., Portland, OR, USA).

### 4.5. Deconvolution and Quantitative Analysis of ^29^Si MAS NMR Spectra

The ^29^Si MAS NMR spectrum of each hydrated sample was analyzed by a method reported by Love et al. [29]. The signal from the unreacted alite phase that obscures the resonances of the early Q^0^(H) and Q^1^ signals was subtracted from the spectrum prior to deconvolution. This was achieved by adjusting the intensity of the ^29^Si MAS NMR spectrum of anhydrous WPC to match the intensity of the Q^0^ signal of alite in the hydrated spectrum. The adjusted background spectrum was then subtracted from the spectrum of the hydrated sample prior to deconvolution using iterative fitting of the Q^0^(H), Q^1^, Q^2^, and Q^2^(1Al) ^29^Si resonances to Voigt lineshapes. The relative abundance of the various Q^n^ species, the degree of hydration, the mean silicate chain length (MCL), and the Al/Si ratio were then calculated from the subtracted and deconvoluted spectra [30,31]. The formulae for the calculations of degree of hydration, MCL, and Al/Si ratio are given below [29,31], as follows:

(1) $$\mathrm{Degree}\;\mathrm{of}\;\mathrm{hydration}\;=\frac{Q^{0}\left(H\right)+Q^{1}+Q^{2}+Q^{2}\left(1\mathrm{Al}\right)}{Q^{0}+Q^{0}\left(H\right)+Q^{1}+Q^{2}+Q^{2}\left(1\mathrm{Al}\right)}\times 100\%$$

(2) $$\mathrm{MCL}=\frac{Q^{1}+Q^{2}+\frac{3}{2}Q^{2}\left(1\mathrm{Al}\right)}{\frac{1}{2}Q^{1}}$$

(3) $$\frac{\mathrm{Al}}{\mathrm{Si}}=\frac{\frac{1}{2}Q^{2}\left(1\mathrm{Al}\right)}{Q^{1}+Q^{2}+Q^{2}\left(1\mathrm{Al}\right)}$$

where Q^n^ represents the intensity of the ^29^Si MAS NMR signal corresponding to the relevant silicate species.

On the assumption that the formation of octameric and hendecameric units in the C–S–H gel structure is negligible during the early stages of hydration, the relative proportions of dimer (D) and pentamer (P) units can be estimated on the basis that D + P = 1, and that MCL = 2D + 5P [24].

### 4.6. Transmission Electron Microscopy

TEM images of WPC, WPC-Bi, and WPC-Zr following hydration for 3 h were obtained by dispersing the sample in methanol prior to deposition onto a carbon film grid. Bright field images were obtained using a JEOL JEM200CX microscope and Gata Orius SC200 digital camera (JEOL, Tokyo, Japan).

## 5. Conclusions

This study followed the early hydration chemistry of white Portland cement blended with 20 wt.% micron-sized bismuth oxide (Bi_2_O_3_) or zirconium oxide (ZrO_2_) by isothermal conduction calorimetry, ^29^Si and ^27^Al magic angle spinning nuclear magnetic resonance spectroscopy, and transmission electron microscopy. Initial and final setting times were also determined by a Vicat apparatus. Bi_2_O_3_ was found to extend the initial and final setting times and retard the degree of hydration by 32% at 24 h. Heat evolution during the acceleration and deceleration phases was reduced and the exotherm arising from renewed ettringite formation was delayed and diminished in the presence of Bi_2_O_3_. In this sample, the mean chain length (MCL) of the C–S–H gel rose from 2.0 to 4.6 between 3 and 24 h as the incorporation of bridging aluminate tetrahedra increased the Al/Si ratio from zero to 0.124. Conversely, ZrO_2_ had no significant impact on either setting time, but was found to accelerate the extent of hydration by 23% within 24 h. Modest increases in heat evolution were attributed to the ‘filler effect’ of the ZrO_2_ particles, as were the enhanced MCL (4.0) and Al/Si ratio (0.101) relative to those of unblended WPC (3.7 and 0.069, respectively) at 24 h. From a clinical perspective, the extended setting times and retarded hydration observed in the presence of bismuth oxide are a distinct disadvantage. Accordingly, the findings of this study indicate that ZrO_2_ is a potentially superior radiopacifying agent to Bi_2_O_3_ in Portland cement-based endodontic restoratives.

## Figures and Tables

**Table 1:** Initial and final setting times for WPC, WPC-Bi and WPC-Zr.

Cement	WPC	WPC-Bi	WPC-Zr
Initial set (min)	143 ± 11 ^a^	255 ± 21 ^b^	163 ± 4 ^a^
Final set (min)	190 ± 1 ^c^	360 ± 14 ^d^	195 ± 10 ^c^

Different superscript letters indicate significant differences at *p* < 0.05.

**Table 2:** Relative abundance of hydrated Q^n^ species, the degree of hydration, the mean silicate chain length (MCL), and the Al/Si ratio of C-S-H gel in WPC, WPC-Bi, and WPC-Zr.

Sample	Time (h)	Q^0^(H) (%)	Q^1^ (%)	Q^2^(1Al) (%)	Q^2^ (%)	Hydration (%)	MCL	Al/Si
WPC	3	0.58	0.47	0.18	0.20	1.4	4.0	0.062
	24	1.41	23.54	5.87	11.44	42.2	3.7	0.069
WPC-Bi	3	2.05	0.15	0.00	0.00	2.2	2.0	0.000
	24	2.54	12.83	7.11	6.13	28.6	4.6	0.124
WPC-Zr	3	0.94	0.66	0.15	0.37	2.1	3.8	0.036
	24	3.57	26.62	10.49	11.19	51.9	4.0	0.101

**Table 3:** Composition of white Portland cement.

Major Oxide Components		Minor Oxide Components		Major Crystalline Phases	
Formula	Mass (%)	Formula	Mass (%)	Formula	Mass (%)
CaO	69.2	MgO	0.49	Ca_3_SiO_5_	65
SiO_2_	25.0	P_2_O_5_	0.43	Ca_2_SiO_4_	22
Al_2_O_3_	1.76	Fe_2_O_3_	0.33	Ca_3_Al_2_O_6_	4.1
SO_3_	2.00	SrO	0.14	Ca_2_(Al/Fe)O_5_	1.0
